# Adding bendamustine to melphalan before ASCT improves CR rate in myeloma vs. melphalan alone: A randomized phase-2 trial

**DOI:** 10.1038/s41409-022-01681-y

**Published:** 2022-04-20

**Authors:** Sarah Farag, Ulrike Bacher, Barbara Jeker, Myriam Legros, Gaelle Rhyner, Jean-Marc Lüthi, Julian Schardt, Thilo Zander, Michael Daskalakis, Behrouz Mansouri, Chantal Manz, Thomas Pabst

**Affiliations:** 1 Department of Medical Oncology, Inselspital, University Hospital and University of Bern, Bern, Switzerland; 2 Department of Hematology, Inselspital, University Hospital and University of Bern, Bern, Switzerland; 3 Center of Laboratory Medicine (ZLM), Inselspital, University Hospital and University of Bern, Bern, Switzerland; 4 Department of Oncology, Cantonal Hospital Fribourg, Fribourg, Switzerland; 5 Regional Hospital Thun, Thun, Switzerland; 6 Bürgerspital Solothurn, Solothurn, Switzerland; 7 Cantonal Hospital Lucerne, Lucerne, Switzerland; 8 ClinaMed, Therwil, Switzerland

**Keywords:** Myeloma, Myeloma

## Abstract

Definite cure remains exceptional in myeloma patients even after high-dose chemotherapy (HDCT) with melphalan (Mel) and autologous stem cell transplantation (ASCT). Thus, improving efficacy of HDCT in MM remains an unresolved issue. This randomized phase II trial compared standard 200 mg/m^2^ Mel HDCT to experimental HDCT with 200 mg/m^2^ bendamustine, given both at days −4 and −3, combined with 100 mg/m^2^ melphalan at days −2 and −1 (BenMel) before ASCT as first-line consolidation in myeloma patients. The primary endpoint aimed to identify at least a 15% improvement in the complete remission rate (stringent CR + CR) after HDCT with BenMel compared with Mel alone. A total of 120 MM patients were 1:1 randomized. The rate of sCR/CR after ASCT was higher in BenMel than in Mel treated patients (70.0% vs. 51.7%; *p* = 0.039). Three patients in the BenMel group (5.0%) had reversible acute renal insufficiency compared with none in Mel patients. Minimal residual disease negativity (<10-5) by flow cytometry was observed in 26 (45.6%) BenMel patients and 22 (37.9%) in the Mel group (*p* = 0.375). Our data suggest that BenMel HDCT is safe and improves the sCR/CR rate compared with standard Mel alone.

## Introduction

High-dose chemotherapy (HDCT) with melphalan at a dose of 200 mg/m^2^ is the standard conditioning regimen since decades in patients with multiple myeloma (MM) who are eligible for autologous stem cell transplantation (ASCT) [1–4]. However, definite cure in MM patients remains exceptional due to residual disease escaping intensive treatment. Consequently, improving the effectiveness of HDCT remains an unmet clinical need [5–9].

Achieving higher response rates after induction treatment and ASCT is supposed to induce longer progression-free (PFS) and overall survival (OS). Improving response rates can be achieved by optimizing induction and/or conditioning and consolidation treatment. Using combinations of different classes of effective compounds together with anti-CD38 treatment in the induction setting before transplant can result in unprecedented response rates and at least some of them have directly translated into improved outcomes following ASCT [10–16].

Others have investigated different HDCT regimens either in combination with melphalan or with other agents to improve the quality of response in MM patients. However, such efforts inevitably faced the problem of increased toxicity of additional cytotoxic compounds such as with busulfan (or others) to HD melphalan suggesting a narrow margin between superior anti-myeloma effectiveness of melphalan-combinations and increased toxicity [17–25].

Bendamustine hydrochloride (Ben) is a cytotoxic compound having both alkylating and antimetabolite properties. It induces extensive DNA damage enhancing single- and double-strand breaks mediating its antineoplastic effect [26–28].

Bendamustine causes mitotic checkpoint inhibition, induces apoptosis through activation of the *TP53* pathway. It has incomplete cross-resistance with other alkylating agents such as melphalan or cyclophosphamide [29, 30]. Finally, bendamustine has an acceptable toxicity profile in patients with MM [17, 31–35].

In a previous study, we investigated dose-intensified bendamustine before tandem transplantation in patients with MM. 200 mg/m^2^ Bendamustine was given for 2 days together with melphalan compared with melphalan HDCT alone. Bendamustine combination regimen was safe with tolerable toxicity. However, acute renal toxicities were more frequently in patients received bendamustine [17].

A single-arm phase II study evaluated the efficacy of 225 mg/m^2^ of bendamustine plus standard dose of melphalan 200 mg/m^2^ conditioning regimen before ASCT in patients with newly diagnosed MM and in relapsed/refractory MM; the study demonstrated favorable safety and encouraging efficacy of the bendamustine combination regimen. Complete remission (CR) was achieved in 51.0% of the patients at D100. The median PFS was 48 and 45 months for patients with newly diagnosed MM and refractory MM [36].

Based on these results, we conducted a randomized, phase II clinical trial to investigate the response rates and toxicities after receiving bendamustine conditioning with melphalan compared with standard HD melphalan before ASCT in patients with MM in first remission.

## Patients and methods

### Patients and study design

This randomized phase II trial aimed to compare bendamustine plus melphalan conditioning to standard-dose melphalan before ASCT. Patients with histologically proven MM (all stages) must have completed first-line induction treatment (or second induction chemotherapy in refractory MM), considered for ASCT in first remission, hematopoietic cell transplantation-specific comorbidity index (HCT-CI) < 6 points and had provided written informed consent. Inclusion criteria were in addition ECOG < 3, creatinine clearance ≥ 40 ml/min, LVEF ≥ 40% within three months prior to start of study medication, and no known pregnancy. In total, 120 patients were randomized 1:1 using RED Cap software to receive HDCT either with melphalan alone (60 patients) or bendamustine plus melphalan (60 patients). Exclusion criteria are outlined in the Supplementary Material.

Two stratification parameters were applied, remission status at registration and creatinine clearance. The study was registered in the WHO International Clinical Trials Registry Platform (ICTRP, http://www.who.int/ictrp/en/; http://clinicaltrials.gov), #: NCT03187223, and in the Swiss National Complementary Database (Portal), SNCTP 000002150 and was approved from Competent Ethics Committee (CEC) Bern (decision number 2016-00442) and the respective regulatory authority Swissmedic (EudraCT #2016-000231-40).

### Study intervention

In the experimental arm (BenMel), patients received bendamustine plus melphalan. Bendamustine was given at a dose of 200 mg/m^2^/day on days −4 and −3. Melphalan was given at dose of 100 mg/m^2^/day on days −2 and −1, followed by ASCT at day 0. In the standard arm (Mel), patients received melphalan at dose of 100 mg/m^2^/day on days −2 and −1, followed by ASCT at day 0. In patients with reduced renal function (creatinine clearance of ≥40 ml/min and <50 ml/min), the dose of bendamustine was reduced to 100 mg/m^2^/day on days −4 and −3, and the dose of melphalan was reduced to 70 mg/m^2^/day, each on days −2 and −1.

Patients were hospitalized for the entire procedure starting with the HDCT until hematologic recovery and clinically sufficient physical reconditioning. Supportive and prophylactic treatments were given according to the policy of the institution. Excessive hydration was mandatory for patients who received bendamustine. All patients received weight-adapted granulocyte colony stimulating factor (G-CSF; filgrastim) starting from day +6 until day +12 after ASCT.

### Post ASCT

All patients with high-risk cytogenetics as well as those with insufficient response (partial remission (PR) or less) after first ASCT (ASCT1) were planned to receive a second ASCT (ASCT2). Patients who received experimental HDCT BenMel for ASCT1 received HDCT Mel for ASCT2. Patients who received HDCT Mel for ASCT1 were offered (and ultimately decided) to receive HDCT BenMel for ASCT2. All patients received Lenalidomide maintenance therapy after ASCT at a dose of 10 mg/day or for 2 years; in the case of GIII/GIV neutropenia and thrombocytopenia, at 5 mg/day. Lenalidomide therapy had to be completely interrupted in four patients due to the development of infections, skin lesions or DRESS syndrome.

### Objectives of the study

The primary objective was to detect an at least 15% improvement in the CR (stringent CR and CR) rate in the experimental combination group (BenMel) compared with the standard melphalan group 60 days after A.

Secondary objectives were to assess acute and late adverse events (AEs) (Common Terminology Criteria for Adverse Events, CTCAE 4.0) during the study period in patients in each treatment group, particularly renal toxicity induced by bendamustine; to assess the hematologic engraftment; and to assess differences in OS and PFS between the two groups.

### Methods

Response rates were assessed according to the International Myeloma Working Group (IMWG) [37]. Bone marrow (BM) was assessed by cytomorphology, histopathology with immunohistochemistry, and immunophenotyping by multiparameter flow cytometry (MFC) for detection of measurable residual disease (MRD). MRD negativity was defined by less than 10^−5^ aberrant plasma cells after measuring at least 1,000,000 nucleated cells.

Clinical assessment and documentation of toxicities exceeding grade II during hospitalization were performed until 60 days after ASCT, with a particular focus on early renal toxicity. Kidney function tests were assessed daily until three days after ASCT or more if clinically indicated Loss >10% of body weight was reported (grade II). Expected AEs were excluded.

Hematological engraftment was assessed daily and calculated from day 0 (day of ASCT) until absolute neutrophil count (ANC) reached >0.5 × 10^9^/L for three consecutive days and until platelet engraftment (platelet > 20 × 10^9^/L) for three days without platelet transfusion. OS was defined as the time from ASCT until death or last follow-up. PFS was defined as the time from ASCT until first relapse or progression, whichever occurred first.

### Statistical analysis

By applying a one-sided significance level of 20%, 60 patients were needed in each group to have 80% power to reject the null hypothesis of no difference between the CR rates of the treatment groups using one sided two-sample proportion test, which had its assumptions met in that case as the two groups were independent and the sample for each group was greater than 30. Sample size calculations were performed using the software package PASS 11. AEs were classified according to number of occurrences, CTCAE grade and type. Chi- square and Fisher’s exact tests were used to detect the difference between the BenMel and Mel groups in terms of causes and types of AEs. We considered *p* values below 0.05 as significant based on non-parametric two-sided statistical tests. Data were summarized as median and range as an estimate of variation for continuous variables, numbers and percentages for categorical variables. Wilcoxon–Mann–Whitney test was used for continuous variables, chi-squared and fisher exact test were used for categorical variables. Variance wasn’t similar between groups across the different continuous metrics of the study. 95% confidence interval (CI) was used. All authors had access to the clinical trial data. PFS and OS were calculated using the Kaplan-Meier method. Statistical analysis was performed using SPSS (IBM Corp., Armonk, NY, USA) and Stata software packages (Stata Corp LP, College Station, TX, USA).

## Results

### Study population

Between 2017 and 2020, 120 patients with MM were randomized. Patients, disease characteristics and treatment regimens are summarized in Table 1.

**Table 1:** Patient and disease characteristics and treatment regimens (Ben/Mel: bendamustine/melphalan).

Parameter	Total cohort	Melphalan	BenMel	*P* value
Number of patients	120	60	60	
Age, median, years (range)	63 (35–74)	62 (35–74)	65 (46–74)	0.200
Gender, male, *n* (%)	76 (63.3)	35 (58.3)	41 (68.3)	0.256
R-ISS stage at diagnosis, *n* (%)				
I	44 (36.7)	23 (39.0)	21 (35.6)	0.701
II	48 (40.7)	26 (44.1)	22 (37.3)	0.449
IIa	28 (58.3)	16 (61.5)	12 (54.5)	0.624
IIb	8 (16.7)	3 (11.5)	5 (22.7)	0.300
II, unclassified	12 (25.0)	7 (26.9)	5 (22.7)	0.738
III	26 (22.0)	10 (16.9)	16 (27.1)	0.179
unknown	2 (1.7)	1 (1.7)	1 (1.7)	1.000
**Myeloma characterization**				
Plasma cell infiltration, bone marrow, median, % (range)	60 (5–100)	60 (10–100)	60 (5–100)	0.649
Calcium >2.75 mmol/l, *n* (%)	16 (14.0)	6 (10.3)	10 (17.9)	0.236
Creatinine >177 μmol/L, *n* (%)	14 (12.7)	5 (9.3)	9 (16.1)	0.262
Hb <100 g/L, *n* (%)	29 (24.8)	11 (19.3)	18 (30.0)	0.137
Abnormal cytogenetics by FISH, *n* (%)	68 (56.7)	33 (55.0)	35 (58.3)	0.715
High-risk cytogenetics by FISH, *n* (%)^a^	17 (25.0)	9 (27.3)	8 (22.9)	0.793
Paraprotein subtype, *n* (%)				
IgG	56 (46.7)	30 (50.0)	26 (43.3)	0.464
IgA	27 (22.5)	12 (20.0)	15 (25.0)	0.511
IgM	1 (0.8)	0 (0.0)	1 (1.7)	0.314
IgD	1 (0.8)	0 (0.0)	1 (1.7)	0.314
Light-chain myeloma	35 (29.1)	18 (30.0)	17 (28.8)	0.433
Osteolytic bone lesions, *n* (%)	96 (80.0)	48 (80.0)	48 (80.0)	1.000
Single lesion	11 (11.5)	7 (14.6)	4 (8.3)	0.118
2 lesions	7 (7.3)	3 (6.2)	4 (8.3)	0.695
>2 lesions	78 (81.2)	38 (79.2)	40 (83.3)	0.601
PET/CT at diagnosis, *n* (%)	58 (49.1)	27 (46.5)	31 (51.7)	0.574
**Previous therapies**				
First induction regimen, *n* (%)	120 (100)	60 (100)	60 (100)	1.000
VRD	94 (78.3)	45 (75.0)	49 (81.7)	0.372
VCD	23 (19.2)	15 (25.0)	8 (13.3)	0.103
VCR	1 (0.8)	0 (0.0)	1 (1.7)	0.310
VDT	1 (0.8)	0 (0.0)	1 (1.7)	0.310
RD	1 (0.8)	0 (0.0)	1 (1.7)	0.310
Second induction regimen, *n* (%)	6 (100.0)	4 (6.8)	2 (3.3)	0.381
VRD	3 (50.0)	2 (50.0)	1 (50.0)	1.000
MP	1 (17.0)	1 (25.0)	0 (0.0)	0.438
CLD	2 (33.0)	1 (25.0)	1 (50.0)	0.559
Bisphosphonates, *n* (%)	95 (79.2)	48 (80.0)	47 (78.3)	0.818
Radiotherapy, *n* (%)	29 (24.1)	18 (30)	11 (18.3)	0.134
**Remission status after induction,** ***n*** **(%)**				
CR	32 (26.9)	18 (30.0)	14 (23.3)	0.408
VGPR	47 (39.2)	22 (36.7)	25 (41.7)	0.574
PR	35 (29.4)	17 (28.3)	18 (30.0)	0.840
SD/PD	6 (5.0)	3 (5.0)	3 (5.0)	1.000

*R-ISS* Revised International Staging System, *VCD* bortezomib, cyclophosphamide, and dexamethasone, *VD* bortezomib and dexamethasone, *CLD* carfilzomib, lenalidomide, and dexamethasone, *VCR* bortezomib, cyclophosphamide, dexamethasone, *MP* melphalan, dexamethasone.

^a^del 13 or 17p, presence of t(4;14) or t(14;16), or amplification of chromosome 1. Other cytogenetic abnormalities are considered standard-risk abnormalities.

The median age was 62 (range, 35–74) and 65 (range, 46–74) years for the melphalan and BenMel groups, respectively (*p* = 0.20). Immunoglobulin G (IgG) was the most frequently involved paraprotein in both groups. In the melphalan group, 33 (55%) patients had known FISH abnormalities vs. 35 (58.3%) patients in the BenMel group (*p* = 0.71). Among them, high-risk cytogenetics were observed in 9 patients (27.3%) vs. 8 (22.9%) patients in the melphalan and BenMel group, respectively (*p* = 0.79).

### Mobilization and stem cell collection

Patients received mobilization chemotherapy with gemcitabine (29.7%; 35 pts) or vinorelbine (39.2%; 47 pts). Finally, 38 (31.7%) patients had only G-CSF without chemotherapy for mobilization. Plerixafor was given in 24 (28.8%) patients, for either one or two days (Supplementary Table 1). The median number of collected CD34+ cells at the day of mobilization was similar in both groups, with 8.29 × 10^6^/kg b.w. in BenMel patients and 8.49 × 10^6^/kg b.w. in patients with melphalan alone.

### Transplantation and engraftment

Because of impaired renal function, four (6.7 %) patients in the BenMel group received reduced doses of HDCT compared with two (3.4%) patients in the melphalan group.

We infused a median number of 3.6 (range 2.1–7.2) × 10^6^ CD34 cells/kg in melphalan patients compared with 3.7 (range 2.3–7.7) × 10^6^ CD34 cells/kg × 10^6^ cells/kg in BenMel patients (Table 2).

**Table 2:** Details of the ASCT and hematopoietic engraftment (BenMel: Bendamustine/melphalan).

Parameters	Total cohort (*n* = 120)	Mel (*n* = 60)	BenMel (*n* = 60)	*P* value
CD34 + cells transplanted, ×10^6^/ kg, median (range)	3.7 (2–7.8)	3.58 (2.1–7.2)	3.67 (2.3–7.7)	0.715
Interval to engraftment, days, median (range)				
Neutrophils >0.5 × 10^9^/L	11 (10–60)	12 (10–18)	11 (10–60)	0.096
Neutrophils >1.0 × 10^9^/L	12 (10–60)	12 (10–35)	11 (10–60)	0.847
Lymphocytes >0.5 × 10^9^/L	13 (9–60)	13 (10–53)	13 (9–60)	0.992
Lymphocytes >1.0 × 10^9^/L	23 (10–143)	25 (10–60)	20 (11–143)	0.109
Platelets >20 × 10^9^/L	12 (9–47)	13 (9–47)	13 (9–30)	0.367
Platelets >50 × 10^9^/L	18 (9–60)	17 (12–53)	19 (9–60)	0.412
Platelets >100 × 10^9^/L	27 (9–102)	27 (14–102)	26 (9–65)	0.937

Time to neutrophil engraftment was defined as the duration between day 0 and the first 3 days of neutrophils >0.5 × 10^9^/L after ASCT. Time to platelet engraftment was defined as the duration between day 0 and the first day of platelets >20 × 10^9^/L after ASCT (without previous platelet transfusion).

The median time until neutrophil engraftment after ASCT was 11 (range 10–60) days in BenMel patients compared with 12 (range 10–18) days in melphalan patients (*p* = 0.096), and the median time until platelet engraftment was 13 days in both groups (*p* = 0.367); thus, all patients had engraftment of both cell lineages with no significant differences (Table 2).

### Hospitalization and treatment-related toxicity

Patients receiving BenMel had longer duration of hospitalization compared with patients with melphalan alone (median 19; range 17–44 days vs. 18; range 11–28 days; *p* = 0.006) (Table 3), which correlated to the longer administration duration of BenMel HDCT. One patient in the BenMel group required prolonged hospitalization (44 days: due to pneumonia).

**Table 3:** Details of infection, transfusion, and duration of hospitalization (BenMel: Bendamustine/melphalan).

Parameter	Total cohort (*n* = 120)	Melphalan (*n* = 60)	BenMel (*n* = 60)	*P* value
Pts with fever ≥38°C, number (% of cohort)	110 (91.7)	51 (85.0)	59 (98.3)	0.008
Median number of febrile episodes (range) per patient	1 (0–2)	1 (0–2)	1 (1–2)	0.977
Fever of unknown causative organism, number (%)	65 (54.2)	36 (60.0)	29 (31.0)	0.2
Infectious microorganism identified, number (% of pts with fever ≥38°C)^a^	55 (50.0)	24 (47.0)	31 (52.5)	0.2
Bacteria (% of germ identified)	51 (92.7)	24 (100.0)	27 (87.0)	0.5
Fungi (% of germ identified)	1 (1.8)	0 (0.0)	1 (3.2)	0.315
Virus (% of germ identified)	11 (20)	0 (0.0)	11 (35.5)	0.001
Pts with PLT transfusion, number (%)	93 (77.5)	45 (75.0)	48 (80.0)	0.336
Pts with RBC transfusion, number (%)	58 (48.7)	28 (47.5)	30 (50.0)	0.544
Transfused PLT, no, median (range)	2 (1–8)	1 (1–5)	2 (1–8)	0.114
Transfused RBCs, no, median (range)	2 (0–6)	1 (0–3)	2 (1–6)	0.793
Hospitalization, days, median (range)	18 (11–44)	18 (11–28)	19 (17–44)	0.006

*PLT* platelets, *RBCs* red blood cells.

^a^Some patients were infected with more than one organism.

AEs are given in Table 4. We documented 56 and 73 AEs in the melphalan and BenMel groups, respectively. The majority of AEs in both arms were grade 3 or less. Only two (3.3%) patients who had received BenMel had grades 4 and 5 AEs. 43 (71.7%) patients who had received HD BenMel developed one or more AE vs. 36 (60.0%) patients who received HD melphalan alone (*p* = 0.18) (Supplementary Table 3).

**Table 4:** Frequencies and types of adverse events (AEs) >GII (BenMel: Bendamustine/melphalan).

AE type, number of pts (%)	Total cohort (*n* = 120)	Melphalan (*n* = 60)	BenMel (*n* = 60)	*P* value
Gastrointestinal disorders	61 (50.8)	31 (51.7)	30 (50.0)	0.8554
Metabolic and nutritional disorders	30 (25.0)	10 (16.7)	20 (33.3)	0.0359
Cardiac and thromboembolic disorders	8 (6.7)	5 (8.3)	3 (5.0)	0.4700
Weight loss >10%	5 (4.2)	0 (0.0)	5 (8.3)	0.0232
Engraftment syndrome	5 (4.2)	3 (5.0)	2 (3.3)	0.6420
Respiratory	4 (3.3)	1 (1.7)	3 (5.0)	0.3117
Nervous system disorders	4 (3.3)	1 (1.7)	3 (5,0)	0.3117
Infection	4 (3.3)	3 (5.0)	1 (1.7)	0.3117
Renal	3 (2.5)	0 (0.0)	3 (5.0)	0.0807
Muscle weakness and fatigue	2 (1.7)	1 (1.7)	1 (1.7)	1.0000
Psychiatric disorders	1 (0.8)	0 (0.0)	1 (1.7)	1.0000
Hearing impairment	1 (0.8)	0 (0.0)	1 (1.7)	1.0000
Fever of unknown origin	1 (0.8)	1 (1.7)	0 (0.0)	0.3168
Total number of AEs	129	56	73	0.187
No of patients with AEs	79 (65.8)	36 (60.0)	43 (71.7)	0.178

Some patients had more than one AE. BenMel: bendamustine/melphalan. The overall chi-squared test showed no significant differences between Mel and BenMel across all AE categories (*P* = 0.187). In addition, all individual categories produced non-significant *p* values for Fisher’s exact test.

Acute renal insufficiency (ARI) occurred in 3 out of 60 (5.0%) BenMel patients, compared with no patient in the melphalan group (*p* = 0.25). ARI was completely reversible with supportive interventions within 6–8 days in all three patients in the BenMel group, and no patient required renal dialysis. Moreover, 42 (70%) patients in the BenMel group had normal serum creatinine at day 60 assessment vs. 54 (90.0%) patients in the melphalan group (*p* = 0.57).

Most patients in both groups had at least one febrile episode. It was more frequently observed in 59 (98.3%) of the BenMel patients compared with 51 (85%) of the melphalan patients (*p* = 0.008). The rate of infection was 51.7% in the BenMel group vs. 40.0% in the Mel group without significant differences (*P* = 0.20). Bacterial infections were preferentially observed with Escherichia coli (E. Coli) and coagulase-negative Staphylococci (Supplementary Table 5). Viral infection occurred in the BenMel group in 11 (35.5%) patients compared with none in the melphalan group (*p* = 0.001) (Table 3). Viral infections were predominantly respiratory viruses including influenza, parainfluenza, rhinovirus and RSV. All patients received acyclovir prophylaxis twice daily 500 mg p.o. until recovery from myelosuppression. Thus, acyclovir prophylaxis was not related to most of the observed viral infections.

Gastrointestinal AEs were common in both treatment arms, which occurred 50% in the BenMel group and 51.7% in the melphalan group without significant differences (*p* = 0.855) (Table 4). Loss of more than 10% of body weight occurred in 5 (8.3%) patients in the BenMel group, but was not detected in the melphalan group (*p* = 0.06)

In the BenMel group, three (5%) patients were admitted to the intensive care unit (ICU) because of acute respiratory distress syndrome (ARDS), septic shock, or pulmonary failure compared with two (3.3%) patients in the melphalan group (due to restrictive cardiomyopathy and pulmonary failure). Finally, treatment-related mortality (TRM) occurred in one (1.6%) patient in the BenMel group due to pneumonia and respiratory failure compared with no patients in the melphalan group.

### Outcomes of ASCT

**Fig. 1:**
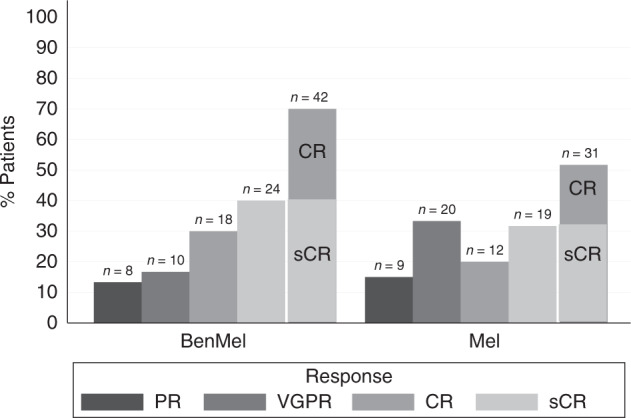
Response rates at day +60 following ASCT in both treatment groups; melphalan (Mel) and BenMel (bendamustine/melphalan) group. PR partial response, VGPR very good partial response, CR complete response, sCR stringent CR.

Figure 1 shows the CR rates by adding HDCT bendamustine to standard dose melphalan. The sCR/CR rate after ASCT before initiation of lenalidomide maintenance treatment was higher in the BenMel group than in the melphalan group (42 patients, 70% vs. 31 patients, 51.7%; *p* = 0.039). Remission rates were sCR in 40% vs. 31.7%; CR in 30.0% vs. 20.0%; very good partial remission in 16.7% vs. 33.3%; and partial remission in 13.3% vs. 15%.

Minimal residual disease negativity assessed in the bone marrow by flow cytometry (defined as <10-5) was observed in 26 (45.6%) patients in the BenMel group compared with 22 (37.9%) in melphalan patients (*p* = 0.38) (Supplementary Table 7). Four patients with PR after ASCT1 in each group received subsequent tandem transplantation.

### Survival

**Fig. 2:**
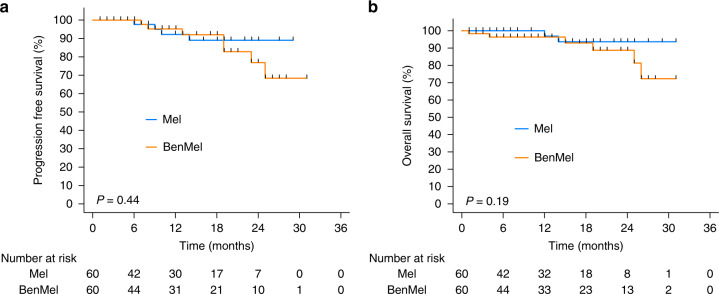
Survival rates comparing high-dose chemotherapy with melphalan with or without bendamustin. **A** Progression-free survival; **B** Overall survival in both treatment groups, Melphalan and BenMel (bendamustine/melphalan). The *x*-axis provides the interval from ASCT.

After a median follow up of 28.7 months, four patients (6.6%) in the melphalan group compared with six patients (10.0%) in the BenMel group had progressed after ASCT. PFS rate at 12 months was 95% in the BenMel group compared with 91% (*p* = 0.551). The OS rate was 96% at 12 months for both groups. The median PFS and OS was not reached in both groups (Fig. 2, A, B). In conclusion, there was no difference in PFS and OS between the two treatment groups (*p* = 0.44 and *p* = 0.19, respectively).

## Discussion

Subsequent HDCT with ASCT after induction chemotherapy is the standard of care for MM patients, who are eligible for ASCT. Despite the increasing armamentarium of chemotherapeutic agents, immunomodulatory drugs (such as lenalidomide and pomalidomide), and antibody treatment (such as elotuzumab, a humanized anti-CS1/SLAMF7 monoclonal antibody, and daratumumab or isatuximab, humanized anti-CD38 monoclonal antibodies), relapse after first-line treatment occurs in most patients. Many trials have tried to improve and prolong the response rates of patients with MM [38–40]. Among them, some trials have studied novel combinations of conditioning regimens before ASCT; such trials faced the problem of added toxicity such as adding busulfan to high-dose melphalan which resulted in veno-occlusive disorders [25, 41].

Bendamustine was investigated in myeloma and lymphoma patients in phase I and II trials, which suggested a tolerable toxicity profile [17, 36, 39, 40]. We and others have reported a higher incidence of acute renal insufficiency (ARI) in patients who had received a bendamustine combination conditioning before either the first or second transplantation compared with standard high-dose melphalan [17].

In the present randomized phase II study, we added bendamustine 200 mg/m^2^ for 2 days to high-dose melphalan 100 mg/m^2^ for 2 days before ASCT in first-line myeloma patients, and we compared this strategy to standard high-dose melphalan. The present trial met its primary endpoint by demonstrating significantly higher rates of sCR/CR after ASCT for the BenMel group compared with the melphalan alone group (70.0% vs. 51.7%; *p* = 0.039). Longer follow-up will clarify whether induction of deep molecular responses after HD BenMel will prolong PFS in those patients.

In our study, acute renal insufficiency (ARI) grade III or higher occurred in 5.0% of all patients in the BenMel group, whereas it was not observed in the melphalan group. Importantly, the three patients with ARI were treated with supportive measures only and required no dialysis, and ARI was fully reversible.

Apart from ARI, gastrointestinal adverse events were the most prevalent in both groups while not being different between the two groups. However, weight loss >10% was only seen in BenMel patients. Fever, mostly due to bacterial infection, was documented in 98.3% patients in the BenMel group as compared with 85.0% in the melphalan group (*p* = 0.008). Patients in the BenMel group had more viral infections (*p* = 0.001) which did not affect or delayed the start of maintenance therapy. Other frequencies and types of adverse events did not differ between the two treatment arms.

After a median follow up of 28.7 months, four patients (6.6%) in the melphalan group and six patients (10.0%) in BenMel group had progressed after ASCT. The PFS rate at 12 months was 95% in the BenMel group compared with 91% (*p* = 0.551). The median PFS was not yet reached for both groups. OS was 96% at 12 months for both groups, and, again, the median OS was not yet reached. The data suggest that differences in survival rates are not yet observed, and longer follow-up will be needed to clarify this issue.

A previous single-arm phase II study added bendamustine 225 mg/m^2^ to standard-dose melphalan 200 mg/m^2^ as a conditioning regimen before ASCT in 35 newly diagnosed and relapsed MM patients. sCR/CR was achieved in 51% of these patients [36, 41], and this rate was lower than observed in our study, which may be related to their smaller cohort size, inclusion of 48% patients with refractory or relapsed myeloma or to the bendamustine dose that was lower than in our study.

In a previous retrospective study, we compared the safety profile of HD BenMel before a second ASCT to melphalan alone before ASCT in 12 patients with refractory or relapsed MM [18]. Acute kidney injuries (grades II and III) following HD BenMel were seen in three (25.0%) patients. Gastrointestinal toxicities were similarly seen after both conditioning regimens, whereas cardiac toxicities were only observed in the group with melphalan [17].

Adding bendamustine to melphalan before ASCT in MM patients appears to be feasible with an acceptable toxicity profile. The most common adverse events in patients receiving bendamustine and melphalan were nausea (94.0%), fatigue (94.0%), hypocalcemia (94.0%), anorexia (91.0%), diarrhea (91.0%), and hypoalbuminemia (91.0%). Febrile neutropenia was seen in 46.0%. Treatment-related mortality occurred in one (1.6%) patient in the BenMel group. Three (5.0%) BenMel patients were admitted to the intensive care unit (ICU) because of acute respiratory distress syndrome (ARDS), septic shock, or pulmonary failure compared with two (3.3%) patients after melphalan alone (because of restrictive cardiomyopathy or pulmonary failure). Longer duration of hospitalization in patients with bendamustine was related to the two additional days needed for HDCT administration. The median time of hospitalization was 19 days for the BenMel group and 18 days for the melphalan patients.

Ours is the first trial prospectively comparing bendamustine and melphalan to melphalan alone before first line ASCT. A previous single arm phase II study reported a median PFS of 47 months after bendamustine and melphalan HDCT, with a 3-year PFS of 78% for newly diagnosed and 57% for relapsed myeloma patients and the 3-year OS was 88% (94% and 81%, respectively) [36]. In our study, the median PFS and OS were not reached given the limited duration of follow-up, and comparisons of survival rates to other studies are not yet possible [36, 42]. The PFS at 12 months in this study was 95% in the BenMel group compared with 91% in the melphalan group (*p* = 0.551), while OS was 96% at 12 months for both groups.

In conclusion, our data indicate that administration of high-dose bendamustine together with melphalan before ASCT in patients with MM is safe. In particular, bendamustine-associated renal toxicity was manageable and reversible in all patients, and hematopoietic engraftment was comparable to standard HDCT with melphalan alone. HDCT with BenMel improves the sCR/CR rate compared with melphalan alone, and may be further explored as a possible new standard in first-line HDCT consolidation for MM patients in first remission.

## Supplementary information

Supplemental Material

Supplementary Figure 1

Patient Flow Chart

Drug Information

Exclusion criteria

## Data Availability

Data are available on request via email from the corresponding author.
