# Supplementary material for: Adding bendamustine to melphalan before ASCT improves CR rate in myeloma vs. melphalan alone: A randomized phase-2 trial
Source: Bone Marrow Transplant. 2022 Apr 20;57(6):990–7. doi: 10.1038/s41409-022-01681-y (PMC9018972; doi:10.1038/s41409-022-01681-y)
Supplement: Supplementary file 1 — Supplemental Material [file 41409_2022_1681_MOESM1_ESM.docx]

**Adding bendamustine to melphalan before ASCT improves CR rate in myeloma vs. melphalan alone: a randomized phase-2 trial**

Online Supplement

Supplementary Tables

Supplementary Table 1: Details of randomization and stratification (BenMel: bendamustine/melphalan).

| **Parameter** | **Total cohort** | **Melphalan** | **BenMel** |
| --- | --- | --- | --- |
| **Valid patients (%)*** | **120 (100.0)** | **60 (100.0)** | **60 (100.0)** |
| Stratification (response rate at the time of randomization) |  |  |  |
| CR or VGPR | 85 (70.8) | 43 (71.7) | 42 (70.0) |
| PR or SD or PD | 35 (29.2) | 17 (28.3) | 18 (30.0) |
| Serum creatinine clearance |  |  |  |
| 40 - 49 ml/min | 5 (4.2) | 3 (5.0) | 2 (3.3) |
| ≥50 ml/min | 115 (95.8) | 57 (95.0) | 58 (96.7) |

*One patient was double randomized (total randomization= 121; valid randomization= 120).

Supplementary Table 2: Details of mobilization and stem cell collection (BenMel: bendamustine/melphalan).

| **Parameter, number of pts (%)** | **Total cohort**  **(n=120)** | **Melphalan**  **(n=60)** | **BenMel**  **(n=60)** |
| --- | --- | --- | --- |
| **Mobilization regimen** |  |  |  |
| Gemcitabine | 35 (29.7) | 19 (31.7) | 16 (26.7) |
| Vinorelbine | 47 (39.2) | 20 (33.3) | 27 (45.0) |
| Growth factors only (no chemotherapy) | 38 (31.7) | 21 (35.0) | 17 (28.3) |
| **Growth factors** |  |  |  |
| G-CSF (Neupogen®), n (%) | 22 (18.5) | 12 (20.0) | 10 (16.9) |
| G-CSF (Filgrastim Teva®), n (%) | 97 (80.8) | 48 (80.0) | 49 (81.7) |
| Plerixafor | 24 (20.0) | 10 (16.7) | 14 (23.3) |
| Plerixafor, single day (+ G-CSF) | 22 (18.3) | 10 (16.7) | 12 (20.0) |
| Plerixafor, two days (+ G-CSF) | 2 (1.7) | 0 (0.0) | 2 (3.3) |
| **Stem cell collection** |  |  |  |
| at 1 day | 106 (88.3) | 53 (88.3) | 53 (88.3) |
| at 2 days | 14 (11.7) | 7 (11.7) | 7 (11.7) |
| Collected frozen cells (×10^6 CD34+ cells/kg), median (range) | 8.38 (2.0 –29.5) | 8.49 (2.0 –29.5) | 8.29 (2.3 –22.9) |

Supplementary Table 3: Frequencies and numbers of adverse events (AEs) in the different treatment groups (BenMel: bendamustine/melphalan).

| **Parameter** | **Total cohort (n=120)** | **Melphalan**  **(n=60)** | **BenMel (n=60)** | **P values** |
| --- | --- | --- | --- | --- |
| **Patients with evidence of one or more AEs, number (%)** | **79 (65.8)** | **36 (60)** | **43 (71.7)** | **0.178** |
| - Patients with 1 AE | 46 (38.3) | 23 (38.3) | 23 (38.3) | 1.000 |
| - with 2 AEs | 20 (16.7) | 8 (13.3) | 12 (20.0) | 0.326 |
| - with 3 AEs | 10 (8.3) | 4 (6.7) | 6 (10.0) | 0.515 |
| - with 4 AEs | 2 (1.6) | 0 (0.0) | 2 (3.3) | 0.157 |
| - with 5 AEs | 1 (0.8) | 1 (1.7) | 0 (0.0) | 0.312 |

Supplementary Table 4. Grades of AEs (adverse events, CTCAE 4.0 grading) and SAEs (severe adverse events) in the treatment groups (BenMel: bendamustine/melphalan).

| **Parameter** | **Total cohort**  **(n=120)** | **Melphalan**  **(n=60)** | **BenMel**  **(n=60)** | **P value** |
| --- | --- | --- | --- | --- |
| **Total number of AEs (%)** | **129 (100)** | **56 (100)** | **73 (100)** | **0.263** |
| **Grades of AEs, number (% of total AEs)** |  |  |  |  |
| Grade II | 3 (2.3) | 0 (0.0)) | 3 (4.1) | 0.44 |
| Grade III | 124 (96.1) | 56 (100) | 68 (93.2) | 0.44 |
| Grade IV | 1 (0.8) | 0 (0.0)) | 1 (1.4) | 0.44 |
| Grade V | 1 (0.8) | 0 (0.0) | 1 (1.4) | 0.44 |
| SAE (hospitalization >24 h) | 23 (17.8) | 10 (17.9) | 13 (17.8) | 0.586 |

**Supplementary Table 5.** Cause of infections in patients in which a causative microorganism was identified (BenMel: bendamustine/melphalan). In some patients, more than one microorganism was identified.

| **Microorganism** | **Total cohort**  **(n=120)** | **Melphalan**  **(n=60)** | **BenMel**  **(n=60)** |
| --- | --- | --- | --- |
| Number of pts with infection due to known causative microorganism (% of the whole cohort) | 55 (45.3%) | 24 (40.0%) | 31 (51.7%) |
| Number of identified microorganisms (% of all identified microorganisms) |  |  |  |
| E. coli | 24 (43.6) | 10 (41.7) | 14 (45.2) |
| Coagulase-negative staphylococcus | 21 (38.1) | 10 (41.7) | 11 (35.5) |
| Enterococcus faecium | 4 (7.2) | 2 (8.3) | 2 (6.5) |
| Streptococcus | 3 (5.5) | 2 (8.3) | 1 (3.2) |
| Vancomycin-resistant enterococcus | 1 (1.8) | 1 (4.2) | 0 (0.0) |
| Staphylococcus | 2 (3.6) | 1 (4.2) | 1 (3.2) |
| Campylobacter jejune | 4 (7.3) | 2 (8.3) | 2 (6.5) |
| Klebsiella pneumoniae | 2 (3.6) | 1 (4.2) | 1 (3.2) |
| Candida albicans | 2 (3.6) | 0 (0.0) | 1 (3.2) |
| Herpes zoster | 1 (1.8) | 0 (0.0) | 1 (3.2) |
| Influenza A, B, or Parainfluenza | 4 (7.3) | 0 (0.0) | 4 (12.9) |
| Respiratory syncytial virus | 3 (5.5) | 0 (0.0) | 3 (9.7) |
| Rhinovirus | 3 (5.5) | 0 (0.0) | 3 (9.7) |

**Supplementary Table 6.** Response rates at day +60 following ASCT (BenMel: bendamustine/melphalan).

| **Parameter, number of pts (%)** | **Total cohort**  **(n=120)** | **Melphalan**  **(n=60)** | **BenMel**  **(n=60)** | **P value** |
| --- | --- | --- | --- | --- |
| sCR + CR | 73 (60.8%) | 31 (51.7) | 42 (70.0) | 0.0397 |
| sCR | 43 (35.8) | 19 (31.7) | 24 (40.0) | 0.3412 |
| CR | 30 (25.0) | 12 (20.0) | 18 (30.0) | 0.2059 |
| VGPR | 30 (25.0) | 20 (33.3) | 10 (16.7) | 0.035 |
| PR | 17 (14.2) | 9 (15.0) | 8 (13.3) | 0.7935 |

Two-sample proportions test was used to measure the differences in each response category between Mel and BenMel; CR, complete remission; PR, partial remission; sCR, stringent complete remission; VGPR, very good partial remission.

**Supplementary Table 7.** Results of minimum residual disease (MRD) by flow cytometry on bone marrow biopsies after ASCT (BenMel: bendamustine/melphalan). MRD results were available in most patients as indicated in the first line.

| **MRD-flow results, number of pts (%)** | **Total cohort (n=115)** | **Melphalan (n=58)** | **BenMel**  **(n=57)** | **P value** |
| --- | --- | --- | --- | --- |
| 10-7 to <10-6 | 38 (33.0) | 16 (27.6) | 22 (38.6) | 0.2095 |
| 10-6 to <10-5 | 10 (8.7) | 6 (10.3) | 4 (7.0) | 0.5266 |
| 10-5 to <10-4 | 27 (23.5) | 13 (22.4) | 14 (24.6) | 0.7859 |
| 10-4 to <10-3 | 28 (24.3) | 15 (25.9) | 13 (22.8) | 0.7027 |
| 10-3 to <10-2 | 11 (9.6) | 8 (13.8) | 3 (5.3) | 0.1199 |
| 10-2 to <10-1 | 1 (0.9) | 0 (0.0) | 1 (1.8) | 0.3110 |

**Supplementary Figures legend**

**Supplemental Figure 1.** Distribution of minimal residual disease (MRD) results by flow cytometry (FCM) on bone marrow samples at day +60 following the ASCT.
