# Supplementary figures and images for: Adding bendamustine to melphalan before ASCT improves CR rate in myeloma vs. melphalan alone: A randomized phase-2 trial
Source: Bone Marrow Transplant. 2022 Apr 20;57(6):990–7. doi: 10.1038/s41409-022-01681-y (PMC9018972; doi:10.1038/s41409-022-01681-y)

Supplementary Figure 1

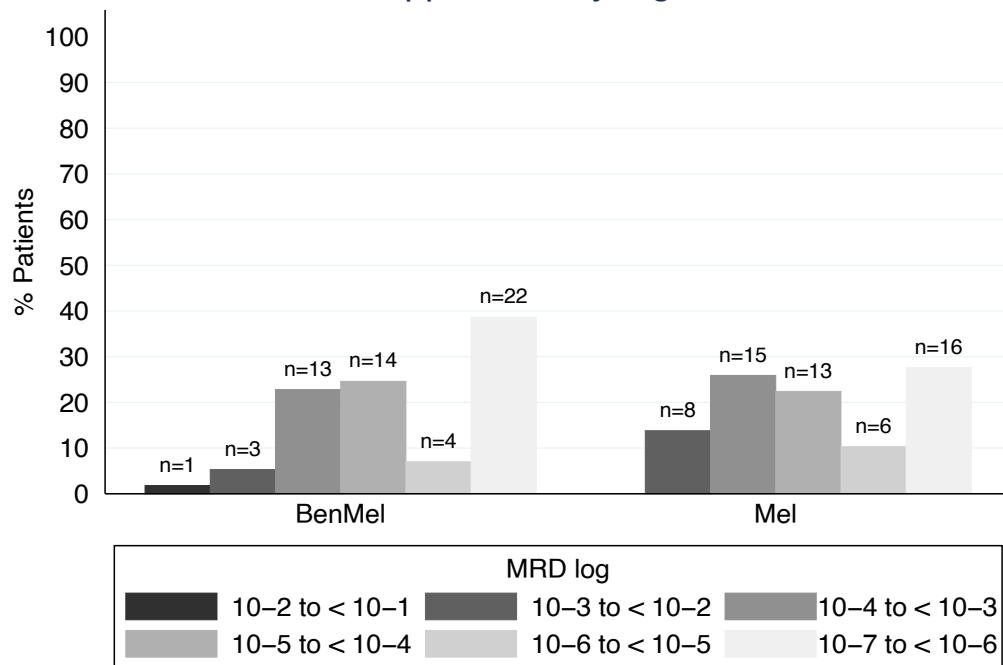

Supplement: Supplementary file 2 — Supplementary Figure 1 [file 41409_2022_1681_MOESM2_ESM.pdf]
