# Supplementary material for: Adding bendamustine to melphalan before ASCT improves CR rate in myeloma vs. melphalan alone: A randomized phase-2 trial
Source: Bone Marrow Transplant. 2022 Apr 20;57(6):990–7. doi: 10.1038/s41409-022-01681-y (PMC9018972; doi:10.1038/s41409-022-01681-y)
Supplement: Supplementary file 3 — Patient Flow Chart [file 41409_2022_1681_MOESM3_ESM.pdf]

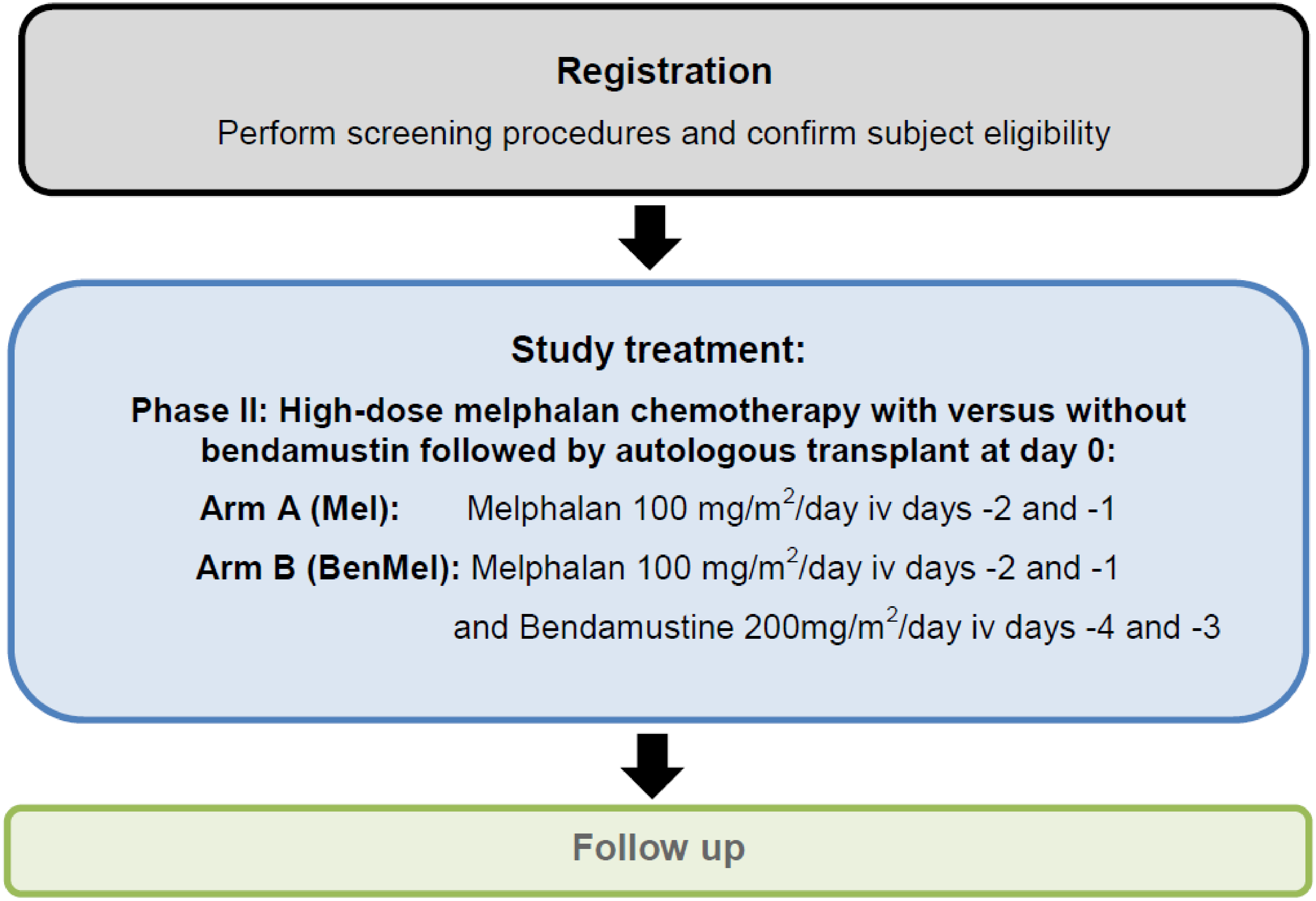

Registration
Perform screening procedures and confirm subject eligibility
Study treatment:
Phase II: High-dose melphalan chemotherapy with versus without bendamustin followed by autologous transplant at day 0:
Arm A (Mel): Melphalan 100 mg/m^2/day iv days -2 and -1
Arm B (BenMel): Melphalan 100 mg/m^2/day iv days -2 and -1
and Bendamustine 200mg/m^2/day iv days -4 and -3
Follow up
