# Supplementary material for: Adding bendamustine to melphalan before ASCT improves CR rate in myeloma vs. melphalan alone: A randomized phase-2 trial
Source: Bone Marrow Transplant. 2022 Apr 20;57(6):990–7. doi: 10.1038/s41409-022-01681-y (PMC9018972; doi:10.1038/s41409-022-01681-y)
Supplement: Supplementary file 4 — Drug Information [file 41409_2022_1681_MOESM4_ESM.docx]

**High-dose bendamustine and melphalan before autologous transplantation improves complete remission rate in myeloma patients**

**compared to melphalan alone**

**Drug information**

**Experimental treatment: high dose Bendamustine plus high dose melphalan (BenMel).**

**Standard treatment: High dose melphalan (Mel).**

1. **Bendamustine:**

**Drug name:** Bendamustine hydrochloride (HCL)

**Trade name:** Ribomustin^®^

**Pharmaceutical form:** Powder for solution for infusion, provided in single brown glass vials containing 100 mg of bendamustine hydrochloride (HCl) powder**.**

**Dose:** 400 mg/m^2^, divided in two doses of 200 mg/m^2^/day on days −4 and −3 before autologous stem cell transplantation (ASCT).

**Dose modification:** In patients with reduced renal function (creatinine clearance of ≥40 mL/min and <50 mL/min), the total dose of bendamustine was reduced to 200 mg/m^2^, divided in two doses of 100 mg/m^2^/day on days −4 and −3.

**Administration**: i.v. infusion, bendamustine powder must be first dissolved in sterile water, then diluted in 500 mL of 0.9% sodium chloride (NaCl) solution and injected over 120 minutes with sufficient hydration before and after administration. At least 4,000 mL of NaCl 0.9% solution should be administered i.v. (2,000 mL before administration with furosemide and 2,000 mL after administration of bendamustine). No discontinuation according to the study protocol.

**Cycle length of BenMel regimen:** The previous dose is given once followed by melphalan on days −2 and −1, and ASCT at day 0.

**Premedication:** Antiemetic drugs are advised (e.g. a 5-HT3 receptor antagonist) and excessive hydration (2,000 mL NaCl 0.9%).

**Supportive medication:** G-CSF (filgrastim) 5 μg/kg b.w. starting at day +6 for a total of seven days after ASCT (or longer if clinically indicated) during neutropenia.

• Platelet infusions are given if platelet levels are <10 G/L, or in case of fever or coagulopathy if platelets are <20 G/L or if clinically indicated.

• Red blood cell transfusions if hemoglobin (Hb) <8 g/dL. RBC and platelet transfusions should be given to maintain a Hb level >8 g/dL and a platelet count >10 G/L, or if clinically indicated.

• Fungal prophylaxis with 400 mg of fluconazole p.o. ought to be given starting at day +1 once per week until recovery from myelosuppression.

• Cotrimoxazole prophylaxis three times a week for 3 weeks after ASCT, and Acyclovir prophylaxis twice daily 500 mg p.o. for three months will be administered to the patients or according to the hospital policy, until recovery from myelosuppression.

**Patient-monitoring parameters for BenMel and Mel arms:**

1. **Screening visit:** Procedures included obtaining written informed consent, measurement of height, weight, and body surface area, pregnancy test, HCTCI score assessment, ECOG score evaluation, echocardiography, blood test (hematology and biochemistry), and myeloma assessment (protein electrophoresis and immunofixation).
2. **ASCT visit (HDCT day until discharge):** Procedures included assessment of time and number of ASCT days, number of CD34+ cells transplanted, infused red blood cell and platelet units, daily complete blood count and biochemistry to assess renal function, number of days of temperature >38.0° and number of febrile episodes, toxicities, and bone marrow assessment.
3. **Post-ASCT visit day 60 assessment (day +30 to +75 after ASCT):** Assessment of ECOG score, hematology, biochemistry, myeloma assessment, acute and late toxicity/AEs (CTCAE 4.03).
4. **Melphalan:**

**Drug name:** Melphalan

**Trade name:** Alkeran^®^

**Dose:** 200 mg/m^2^, divided in two doses of 100mg/m^2^/day on days -2 and -1 followed by ASCT.

**Dose modification:** For patients with decreased renal function defined as a creatinine clearance ≥40 mL/min and <50 mL/min, melphalan will be reduced to 70 mg/m^2^/day at days -2 and -1.

**Administration**: Dose of 100 mg/m^2^/day, will be administered as i.v. infusion diluted in 500 mL of NaCl 0.9% solution over 60 minutes at days -2 and -1 before ASCT (day 0). At least 4,000 mL of NaCl 0.9% solution should be administered i.v. (2,000 mL before administration with furosemide and 2,000 mL after administration of melphalan). No discontinuation according to the study protocol.

**Cycle length:** The previous dose is given once before ASCT at day 0.

**Premedication:** Antiemetic drugs are advised (e.g. a 5-HT3 receptor antagonist), KCL and excessive hydration (4,000 mL NaCl 0.9%).

**Supportive medication:**

- G-CSF (filgrastim) 5 μg/kg b.w. starting at day +6 for a total of seven days after ASCT (or longer if clinically indicated) during neutropenia.
- Platelet infusions are given if platelet levels are <10 G/L; or in case of fever or coagulopathy if platelets are <20 G/L or if clinically indicated.
- Red blood cell transfusions if hemoglobin (Hb) <8 g/dL. Packed RBCs and platelet transfusions should be given to maintain a Hb level >8 g/dL and platelet counts >10 G/L, or if clinically indicated.
- Fungal prophylaxis with 400 mg of fluconazole p.o. ought to be given starting at day +1 once per week until recovery from myelosuppression.
- Cotrimoxazole prophylaxis three times a week for 3 weeks after ASCT, and Acyclovir prophylaxis twice daily 500 mg p.o. for three months will be administered to the patients or according to the hospital policy, until recovery from myelosuppression.
