# Supplementary material for: Adding bendamustine to melphalan before ASCT improves CR rate in myeloma vs. melphalan alone: A randomized phase-2 trial
Source: Bone Marrow Transplant. 2022 Apr 20;57(6):990–7. doi: 10.1038/s41409-022-01681-y (PMC9018972; doi:10.1038/s41409-022-01681-y)
Supplement: Supplementary file 5 — Exclusion criteria [file 41409_2022_1681_MOESM5_ESM.docx]

**Adding bendamustine to melphalan before ASCT improves CR rate in myeloma vs. melphalan alone: a randomized phase-2 trial**

**Exclusion criteria**

- Patients with uncontrolled acute infection.
- Patients with a transplantation comorbidity index (HCTCI) > 6 points (HCTCI may be postponed to the treatment visit).
- Patients with concurrent malignant disease with the exception of basalioma/spinalioma of the skin or early-stage cervix carcinoma, or early-stage prostate cancer. Previous treatment for other malignancies (not listed above) must have been terminated at least 24 months before registration and no evidence of active disease shall be documented since then.
- Patients with major coagulopathy or bleeding disorder.
- Patients with other serious medical condition that could potentially interfere with the completion of treatment according to this protocol or that would impair tolerance to therapy or prolong hematological recovery.
- Patients with lack of cooperation to allow study treatment as outlined in this protocol.
- Pregnancy or lactating female patients.
- The use of any anti‐cancer investigational agents within 14 days prior to the expected start of trial treatment
- Patients with contraindications and hypersensitivity to any of the active chemotherapy compounds.
